# Towards a More Individually Tailored Exercise Prescription for Promoting Cardiovascular Health

**DOI:** 10.3390/jcdd9110401

**Published:** 2022-11-18

**Authors:** Giuseppe Caminiti, Ferdinando Iellamo

**Affiliations:** 1 Department of Rehabilitation Cardiology, IRCCS San Raffaele Pisana, 00163 Rome, Italy; 2 Department of Human Science and Promotion of Quality of Life, San Raffaele Open University, 00166 Rome, Italy; 3 Department of Clinical Science and Translational Medicine, University of Tor Vergata, 00133 Rome, Italy

## 1. Introduction

The beneficial effects of exercise training (ET) in promoting cardiovascular health have been well established [1]. Regular physical exercise is associated with a decreased risk of developing cardiovascular disease (CVD) and reduced cardiovascular mortality [2]. However, the development of ET as an effective therapeutic weapon for preventing and treating CVD is ongoing since many questions regarding ET and cardio-protection remain unanswered. For example, the intensity and volume of ET needed to optimize cardiovascular benefits in different clinical contexts remain uncertain; similarly, several mechanisms through which ET carries out its cardiovascular effects are still unclear; lastly, in order to develop an increasingly individually tailored ET prescription, further studies that include specific populations underrepresented in previous trials are needed. This Special Issue collects seven original articles and three reviews evaluating the effects of ET in a wide range of clinical conditions: four out of ten papers deal with ET in chronic heart failure (CHF) [3,4,5,6]; two with benefits of ET in subjects with congenital heart disease (CHD) [7,8]; two with blood pressure (BP) responses to acute or long-term exercise [9,10]; one with cardiovascular response to resistance exercise (RE) in elderly [11] and one with clinical consequences of coronavirus disease (COVID-19) in athletes [12].

## 2. Exercise and Chronic Heart Failure

ET is strongly recommended for patients with CHF since it improves exercise tolerance and quality of life [3]. The use of moderate continuous training is supported by robust evidence and represents the gold standard for these patients [13,14]; however, high-intensity exercise and RE can produce additional benefits [15]; therefore, the strategy of adopting a more individualized approach in exercise prescription, pursuing the goal of maximizing the benefits in each patient is now widely adopted [16]. Karatzanos et al. [3] tested the acute cardiorespiratory response of CHF patients to single exercise sessions at three different intensities: high-intensity interval, high-intensity continuous, and low-intensity continuous exercises. They found similar increases in oxygen consumption at peak exercise (VO_2_ peak) between high-intensity intervals and high-intensity continuous exercises, while low-intensity continuous exercise elicited more modest VO_2_ peak increases. Since VO_2_ peak is a strong prognostic parameter in CHF, developing ET protocols with the greatest impact on VO_2_ peak is a primary goal for physicians. Therefore, the results obtained by Karatzanos et al. deserve further validation through long-term comparisons between different ET protocols. The effects of ET in CHF have been assessed mainly in patients with reduced ejection fraction (HFrEF). However, heart failure with preserved ejection fraction (HFpEF) represents the most common HF phenotype in the elderly population, and its prevalence is expected to increase in the near future [17]. HFpEF is a separate clinical condition whose management is a challenge for physicians, considering the scarcity of evidence-based treatments available. Although many studies have investigated the potential benefits of ET in HFpEF in the last decade, there are still several unclear points on this topic. Crisci et al. [4] published a comprehensive overview of the effects of ET in patients with HFpEF, providing an update of current knowledge in this research area. The trials included in this review agree by showing significant improvements in cardiorespiratory function after ET. ET benefits seem to occur through peripheral mechanisms since central systolic and diastolic functions did not significantly change after ET. Clearly, further investigations are needed to fully establish the spectrum of effects produced by ET in HFpEF and to better understand the mechanisms by which ET exerts its effects in these patients. Among CHF patients, those with an implantable cardiac device (ICD) are often characterized by an advanced stage of the disease, poor clinical conditions, and muscle wasting. Their limited exercise tolerance and the fear of receiving an ICD shock with elevated heart rates during exercise have led to a substantial undertraining of these patients. In most studies, patients with ICD were trained with low- or moderate-intensity aerobic exercises, while RE has been used sporadically [18]. Squeo et al. [5] presented the results of the FIDE Project (Fitness Implantable DEvice); the effects of a combined training protocol, including both aerobic and RE, in patients with ICD were assessed. The study recruited 30 subjects undergoing an 8-weeks ET program consisting of combined ET plus flexibility exercises. The patients performed two training sessions per week. Interestingly all patients completed the training protocol without adverse cardiac events or ICD shocks. At the end of the training program, improvements in VO_2_ peak of 2.0 mL/kg/min were observed, along with significant improvements in muscular strength. These results are promising, and further studies, including direct comparisons between combined training and continuous aerobic training are needed to assess the advantages (if any) of prescribing combined training rather than continuous aerobic training alone in patients with ICD. Autonomic nervous system imbalance is among the most important pathophysiological aspects of CHF; it contributes to the progression of the disease and is a marker of exercise intolerance and unfavorable prognosis. The benefits of ET in CHF are at least in part due to its modulatory effects on sympathetic over-activation [19]. The methodological review of Iellamo et al. [6] focused on two available techniques for the assessment of parasympathetic and sympathetic nerve function, heart rate variability (HRV), and iodine-123 metaiodobenzylguanidin (MIBG). The review underlines that HRV and MIBG provide complementary information on autonomic nervous system function. On this basis, it provides a rationale for the combined use of these two techniques in CHF patients. In particular, the authors suggest that the concomitant use of HRV and MIBG in CHF patients undergoing exercise training could permit a better understanding of the mechanisms underlying the autonomic benefits of ET in CHF.

## 3. Exercise and Congenital Heart Disease

The population of adults living with congenital heart disease (CHD) has grown rapidly in recent decades due to major advances in surgical and medical care. Although exercise intolerance is a key symptom of CHD and, despite improvements in VO_2_ peak after ET have been described in these patients [20], the therapeutic role of ET for CHD remains undefined, and formal exercise advice is not routinely provided for these patients. Among patients with CHD, adults with Hypoplastic Left Heart Syndrome (HLHS) constitute a small subgroup characterized by a marked exercise intolerance that severely impairs their quality of life. Because HLHS is a relatively rare condition, until now, no studies have investigated the effects of ET in patients carrying this condition. Perrone et al. [7] published the first pilot study on this topic: they evaluated the effects of a short ET protocol in HLHS. The study included 12 subjects who performed three sessions per week for 4 weeks. At the end of the training program, improvements in cardiorespiratory performance and cardiac biomarkers were observed. This very promising result deserves further confirmation in trials and comparison to no exercise. Patients with repaired Tetralogy of Fallot may experience long-term hemodynamic consequences related to pulmonary regurgitation or residual right ventricular outflow tract obstruction that hesitate in right ventricular volume/pressure overload and ultimately can lead to congestive heart failure. The study of Leonardi et al. [8] deepens the relationship between right ventricular hemodynamic changes and exercise intolerance in asymptomatic adults with repaired Tetralogy of Fallot. Interestingly they observed that these patients, though asymptomatic, had reduced VO_2_ peaks compared to the control. This study has two main points: it contributes to better understanding the mechanisms underlying exercise intolerance in adults with repaired Tetralogy of Fallot and to develop specific ET protocols for these patients; moreover, it underlines the key role of cardiopulmonary testa for identifying, among asymptomatic patients, those who can benefit the most from ET interventions.

## 4. Exercise and Blood Pressure Variability and Reactivity

An elevated short-term blood pressure variability (BPV) is recognized as a consistent risk factor associated with target organ damage and increased cardiovascular events independent of hypertension [21]. ET, in particular aerobic exercise, is a recognized non-pharmacological intervention for lowering blood pressure (BP) in hypertensive subjects and for containing BP increases in pre-hypertension [22]. Conversely, its role in reducing short-term BPV is still debated. Concurrent training, including in the same session aerobic and resistance exercises, has proved more effective than continuous aerobic training alone in reducing short-term BPV in hypertensive males [23], but similar studies failed to show any effect on blood pressure variability in women [24,25]. Caminiti et al. [9] first demonstrated the effectiveness of concurrent training in reducing short-term BPV in hypertensive women. Interestingly the authors obtained this result by increasing the training intensity regarding the resistance component of the sessions. The results of this study are promising but need further confirmation in larger trials. BP increases to a given stress stimulus in relation to baseline values has been referred to as BP reactivity. An exaggerated BP reactivity is typically observed in hypertensive subjects, while in the general population, it is associated with the development of hypertension and CVD [26]. While vast literature shows that BP response to stressors is attenuated after a single session of aerobic exercise [27], cardiovascular responses to other exercise modalities are much less documented. Chen et al. [10] performed a comprehensive review of studies evaluating the effectiveness of single sessions of different exercise modalities in modulating the cardiovascular response to stressors. Two main aspects emerge from this review, and they both concern current limitations: considering the small number of studies including subjects with hypertension or at high risk of CVD, the BP response to stressors is much less characterized in these patients compared to healthy subjects. Secondly, when compared to the results of different studies, problems arise due to huge variability in exercise intensities and the volumes adopted in different protocols.

## 5. Miscellaneous

In older people, RE increases muscle strength, prevents decline in skeletal muscle mass, and positively impacts daily life activities. Although a minimum intensity of 60% to 70% of 1 repetition maximum (1RM) is considered necessary for improving muscle strength and inducing muscle hypertrophy [28], in specific populations, benefits have also been obtained with lower RE intensities, particularly in association with blood flow restriction [29]. Amorin et al. [11] evaluated the effectiveness and safety of a single session of low-intensity blood flow restriction RE (at 20% of 1-RM) compared with RE performed at 60% of 1-RM in octogenarian patients. They found that both intensities were well tolerated, and no side effects occurred during the sessions. Moreover, they observed an increase in arterial stiffness that was similar in the two groups. More studies are needed to establish the long-term benefits of low-intensity blood flow restriction RE in very elderly subjects; and the long-term compliance that can be obtained with this exercise modality. The COVID-19 pandemic has profoundly impacted athletics, and the question of safely resuming competitive sports after being infected with SARS-CoV-2 has been a source of significant debate. The study of Casasco et al. [12] assessed the prevalence of abnormal return-to-play tests according to the Italian Federation of Sport Medicine database. Overall, they found that cardiac complications from COVID-19 were rare, with more than 98% of competitive athletes being asymptomatic or mildly symptomatic. Myocarditis was confirmed in five athletes (0.12% of the total population), which is in line with similar reports [30]. The study contributes to clarifying the epidemiological burden of cardiovascular involvement during SARS-CoV-2 infection in athletes and helps develop effective diagnostic protocols for a safe return to play.

## 6. Conclusions

In conclusion, the findings of the original articles and reviews of this Special Issue contribute to expanding the indications of ET in the field of cardiovascular protection. At the same time, they move in the direction of a more individually tailored ET prescription.
